# Supplementary material for: The Evolution and Characterization of the RNA Interference Pathways in Lophotrochozoa
Source: Genome Biol Evol. 2024 May 7;16(5):evae098. doi: 10.1093/gbe/evae098 (PMC11114477; doi:10.1093/gbe/evae098)
Supplement: evae098_Supplementary_Data [file evae098_supplementary_data.zip › Supplementary_materials.zip!/selected_miRNAs/NoGe_selected_miRNAs/mirdeep_pdf_files/NMRB01000152.1_Notospermus_geniculatus_strain_Ushimado_scaffold152,_whole_genome_shotgun_sequence_5838.pdf]

Provisional ID : NMRB01000152.1_Notospermus_geniculatus_strain_Ushimado_scaffold152,_whole_genome_shotgun_sequence_5838
Score total : 23319.1
Score for star read(s) : 3.9
Score for read counts : 23310.4
Score for mfe : 1.8
Score for randfold : 0
Score for cons. seed : 3
Total read count : 45734
Mature read count : 45719
Loop read count : 0
Star read count : 15

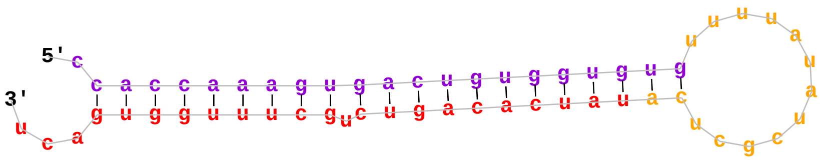

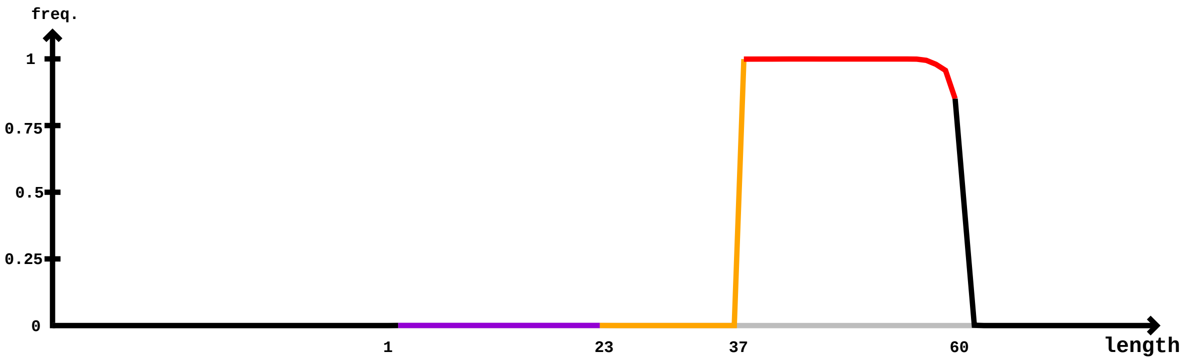

Star Mature

```
5'- ccacaccuugcucuucuccuacgugaccaucgagccaccaaagugacuguggugugguuuuauaucgcucauaucacagucugcuuuggugacuugaauguguucacucaaacc -3' obs
    ccacaccuugcucuucuccuacgugaccaucgagccaccaaagugacuguggugugguuuuauaucgcucauaucacagucugcuuuggugacuugaauguguucacucaaacc      exp
    ......................(((((((((((.((((((((((((((((((((((............)))))))))))).))))))))).)))).))).).))))....... reads mm sample
    ..................................ccaccaaagugacuguggugug......................................................... 1 0 Mm3
    ..................................ccaccaaagugacuguggugugguuuuauaucAcuc............................................ 1 1 Mm3
    ........................................................uuuuauaucAcucauaucacag................................... 1 1 Mm3
    .....................................................................Cuaucacagucugcuuuggugacu.................... 1 1 Mm3
    ......................................................................uaucacagucugcuuugg......................... 1 0 Mm3
    ......................................................................uaucacagucugcuuuggu........................ 11 0 Mm3
    ......................................................................uaucacagucAgcuuuggu........................ 1 1 Mm3
    ......................................................................uaucacagucugcuuuggug....................... 23 0 Mm3
    ......................................................................uaucacagucugcuuugguU....................... 1 1 Mm3
    ......................................................................uaucacagucugcuuugAug....................... 1 1 Mm3
    ......................................................................uaucacagucugcuuugguA....................... 1 1 Mm3
    ......................................................................uaucacagucugcAuuggug....................... 1 1 Mm3
    ......................................................................uaucacagucugcuuugAuga...................... 2 1 Mm3
    ......................................................................uaucacagucugcuuuggugG...................... 12 1 Mm3
    ......................................................................uaucacagucugcuuuggugU...................... 1 1 Mm3
    ......................................................................uaucacagucugcuuuggugа...................... 72 0 Mm3
    ......................................................................uaucacagucugcuuugCuga...................... 1 1 Mm3
    ......................................................................uaucacagucugcuuugAugac..................... 2 1 Mm3
    ......................................................................uaucacagucugcuuuggugUc..................... 1 1 Mm3
    ......................................................................uaucacagucugcuuugUugac..................... 3 1 Mm3
    ......................................................................uaucacagucugcuuugguAac..................... 1 1 Mm3
    ......................................................................uaucacagucugcuuugCugac..................... 4 1 Mm3
    ......................................................................uaucacagucugcuuuggugac..................... 428 0 Mm3
    ......................................................................uaucacagucugcuuuUgugac..................... 1 1 Mm3
    ......................................................................uaucacagucugcuuugAugacu.................... 12 1 Mm3
    ......................................................................uaucaNagucugcuuuggugacu.................... 1 1 Mm3
    ......................................................................Aaucacagucugcuuuggugacu.................... 1 1 Mm3
    ......................................................................uaucacagucugcCuuggugacu.................... 1 1 Mm3
    ......................................................................uaucacagucugcuuugCugacu.................... 11 1 Mm3
    ......................................................................uaAcacagucugcuuuggugacu.................... 1 1 Mm3
    ......................................................................uaucacagucugcuuAggugacu.................... 1 1 Mm3
    ......................................................................uaucacagucugcuuuggugacA.................... 9 1 Mm3
    ......................................................................uaucacagucugcuuuUgugacu.................... 5 1 Mm3
```

Star / Mature			
ccacaccuugcucuucuccuacgugaccaucgagccaccaaagugacugugguguguuuuauaucgcucauaucacagucugcuuuggugacuugaauguguucacucaaacc			
....uaucCcagucugcuuuggugacu....................	1	1	Mm3
....uGucacagucugcuuuggugacu....................	1	1	Mm3
....Naucacagucugcuuuggugacu....................	1	1	Mm3
....uaucacagucugcuuuggugacC....................	2	1	Mm3
....uaucacagucugcuuuggugUcu....................	1	1	Mm3
....uaCcacagucugcuuuggugacu....................	1	1	Mm3
....uaucacagucugcuuuggugacu....................	2495	0	Mm3
....uauUacagucugcuuuggugacu....................	2	1	Mm3
....uaucacaAucugcuuuggugacu....................	1	1	Mm3
....uaucacagucugcuAuggugacu....................	1	1	Mm3
....uaucacagucugcuuuCgugacu....................	8	1	Mm3
....uaucacGgucugcuuuggugacu....................	4	1	Mm3
....uaucacagucugcuuugUugacu....................	12	1	Mm3
....uaucacagucugcuuuggGgacu....................	1	1	Mm3
....uaucacagucugcuuuggugacG....................	64	1	Mm3
....uaucacagucugcAuuggugacu....................	1	1	Mm3
....uaucacagucugcuuuAgugacu....................	6	1	Mm3
....uaucacagucCgcuuuggugacu....................	3	1	Mm3
....uaucacagCcugcuuuggugacu....................	1	1	Mm3
....uaucacagucAgcuuuggugacu....................	5	1	Mm3
....uaucacagucugUuuuggugacu....................	1	1	Mm3
....uaucaUagucugcuuuggugacu....................	2	1	Mm3
....uaucacagucugcuuuggugacuA...................	2	1	Mm3
....uaucacagucugcuuuggugacuu...................	65	0	Mm3
....uCucacagucugcuuuggugacuu...................	1	1	Mm3
....uaucacagucugcuuuggugacuG...................	1	1	Mm3
....uaucacagucugcuuuggugacuuU..................	9	1	Mm3
....uaucacagucugcuuuggugacuAg..................	1	1	Mm3
....ucacagucugcuuuggugacu....................	4	0	Mm3
....acagucugcuuuggugacu....................	1	0	Mm3
....Cuaucacagucugcuuuggugacu....................	2	1	Fm3
....uaucacagucugcuuugg.........................	7	0	Fm3
....uaucacagucugcuuugAu........................	2	1	Fm3
....uaucacagucugcuuugUu........................	1	1	Fm3
....uaucacagucugcuuuggA........................	5	1	Fm3
....uaucacagCcugcuuuggu........................	1	1	Fm3
....uaucacagucugcuuuggu........................	136	0	Fm3
....uaucacagucugcuuuAgu........................	1	1	Fm3
....uaucacagucugcuuugAug.......................	5	1	Fm3
....uaucacagucugcuuugguU.......................	28	1	Fm3
....uaucacGgucugcuuuggug.......................	6	1	Fm3
....Aaucacagucugcuuuggug.......................	2	1	Fm3
....uaucacagucugcuuugCug.......................	2	1	Fm3
....uaucacagucugcuuugUug.......................	2	1	Fm3
....uaucacagucugcuuugguA.......................	26	1	Fm3
....Naucacagucugcuuuggug.......................	1	1	Fm3
....uaucacagucugcuuuAgug.......................	1	1	Fm3
....uaucacagucugcuuugguC.......................	1	1	Fm3
....uaucacagucugcuuuggug.......................	426	0	Fm3
....uaucacagCcugcuuuggug.......................	1	1	Fm3
....uaucacagucugcuuugAuga......................	4	1	Fm3
....uaucacagucugcuuugCuga......................	1	1	Fm3
....uaucacagucugcuuuggugU......................	27	1	Fm3
....uaucacagucugcuuuggugа......................	508	0	Fm3
....uaucacagucugcuuuUguga......................	1	1	Fm3
....uaucacagucugcuuugUuga......................	2	1	Fm3
....uaucacagucugcuuuggugac.....................	2294	0	Fm3
....uaucacGgucugcuuuggugac.....................	23	1	Fm3
....uaucacagucugcuuuggCgac.....................	1	1	Fm3
....uaucacagucCgcuuuggugac.....................	4	1	Fm3
....uaucacagucuCcuuuggugac.....................	1	1	Fm3
....uaucacaUucugcuuuggugac.....................	1	1	Fm3
....uaucacagucugcuuuggugaU.....................	5	1	Fm3
....uaucacagucugcuuugguAac.....................	1	1	Fm3
....uaucacagucGgcuuuggugac.....................	3	1	Fm3
....uaCcacagucugcuuuggugac.....................	4	1	Fm3
....uaucacaAucugcuuuggugac.....................	1	1	Fm3
....uaucacagucugcuuugCugac.....................	8	1	Fm3
....uauGacagucugcuuuggugac.....................	1	1	Fm3

```
                                      Star                                 Mature
ccacaccuugcucuucuccuacgugaccaucgagccaccaaagugacuguggugugugUuuuauaucgcucauaucacagucugcuuuggugacuugaauguguucacucaaacc
........................................................................uUucacagucugcuuuggugac.....................	1	1	Fm3
........................................................................uaucacagucugcuuuUgugac.....................	4	1	Fm3
........................................................................uaucacagucugcuuugUugac.....................	9	1	Fm3
........................................................................uaucacagucugcuuugguUac.....................	2	1	Fm3
........................................................................uaucacagucugcuuugAugac.....................	12	1	Fm3
........................................................................uaucacagucugUuuuggugac.....................	1	1	Fm3
........................................................................uaucacCgucugcuuuggugac.....................	1	1	Fm3
........................................................................uaucacagucugcuuuAgugac.....................	5	1	Fm3
........................................................................uaucacagucAgcuuuggugac.....................	4	1	Fm3
........................................................................uGucacagucugcuuuggugac.....................	1	1	Fm3
........................................................................uaucacagucugcuuCggugac.....................	1	1	Fm3
........................................................................uaucacagucugcuuuCgugac.....................	5	1	Fm3
........................................................................uaucacagucuAcuuuggugacu....................	2	1	Fm3
........................................................................uaucacagucAgcuuuggugacu....................	37	1	Fm3
........................................................................uauGacagucugcuuuggugacu....................	2	1	Fm3
........................................................................uaucacagucugcuuuggugacC....................	20	1	Fm3
........................................................................uaucGcagucugcuuuggugacu....................	2	1	Fm3
........................................................................uaucacagucugcuuuggAgacu....................	2	1	Fm3
........................................................................uaucacUgucugcuuuggugacu....................	2	1	Fm3
........................................................................uaucCcagucugcuuuggugacu....................	5	1	Fm3
........................................................................uaucacagucugcuuuggugCcu....................	1	1	Fm3
........................................................................uaucacagucugcGuuggugacu....................	1	1	Fm3
........................................................................uaucacagucugcuuuCgugacu....................	31	1	Fm3
........................................................................uaGcacagucugcuuuggugacu....................	4	1	Fm3
........................................................................uaucacCgucugcuuuggugacu....................	1	1	Fm3
........................................................................uaucacagAcugcuuuggugacu....................	2	1	Fm3
........................................................................uaucacaguUugcuuuggugacu....................	13	1	Fm3
........................................................................uauAacagucugcuuuggugacu....................	4	1	Fm3
........................................................................uaucacagucugcuuugguUacu....................	2	1	Fm3
........................................................................uaucUcagucugcuuuggugacu....................	2	1	Fm3
........................................................................uaucacagCcugcuuuggugacu....................	5	1	Fm3
........................................................................uaucacagucugcuuuggugaAu....................	1	1	Fm3
........................................................................uaucacagucugcuuuggugacu....................	16247	0	Fm3
........................................................................uaucacagucugcuuCggugacu....................	3	1	Fm3
........................................................................uUucacagucugcuuuggugacu....................	3	1	Fm3
........................................................................Caucacagucugcuuuggugacu....................	4	1	Fm3
........................................................................uaucacagucugcuuuggugUcu....................	1	1	Fm3
........................................................................uaucacagucGgcuuuggugacu....................	19	1	Fm3
........................................................................uaucacagucugcuuuggCgacu....................	1	1	Fm3
........................................................................uaucacagucugcCuuggugacu....................	10	1	Fm3
........................................................................Aaucacagucugcuuuggugacu....................	2	1	Fm3
........................................................................uaucacagucugcuuuggugacG....................	17	1	Fm3
........................................................................uaucacagucCgcuuuggugacu....................	13	1	Fm3
........................................................................uaucacaUucugcuuuggugacu....................	4	1	Fm3
........................................................................uaucaUagucugcuuuggugacu....................	9	1	Fm3
........................................................................uaucacagucugcAuuggugacu....................	5	1	Fm3
........................................................................uaucacGgucugcuuuggugacu....................	343	1	Fm3
........................................................................uaucacagucugcuAuggugacu....................	1	1	Fm3
........................................................................uaucacaguAugcuuuggugacu....................	5	1	Fm3
........................................................................uaucaNagucugcuuuggugacu....................	5	1	Fm3
........................................................................uaucacagucugcuuuAgugacu....................	49	1	Fm3
........................................................................uaucacagucugcuuuggugacA....................	31	1	Fm3
........................................................................uaucacagucugcuuAggugacu....................	3	1	Fm3
........................................................................uaucacagucugcuuugAugacu....................	96	1	Fm3
........................................................................uaucacagucugcuuugguCacu....................	4	1	Fm3
........................................................................uaucacagucugcuuGggugacu....................	2	1	Fm3
........................................................................uaucacagucugcuuugUugacu....................	66	1	Fm3
........................................................................uaucacagucugcuuuggugaUu....................	1	1	Fm3
........................................................................uaucacagucugcuuugCugacu....................	99	1	Fm3
........................................................................uGucacagucugcuuuggugacu....................	4	1	Fm3
........................................................................uaucacagucugcuuuUgugacu....................	31	1	Fm3
........................................................................uCucacagucugcuuuggugacu....................	2	1	Fm3
........................................................................uaucacagucugcuuugguAacu....................	5	1	Fm3
........................................................................uaAcacagucugcuuuggugacu....................	4	1	Fm3
........................................................................uaucacagucugAuuuggugacu....................	3	1	Fm3
........................................................................uaucacagucugUuuuggugacu....................	26	1	Fm3
........................................................................uaucacaguGugcuuuggugacu....................	1	1	Fm3
........................................................................uauUacagucugcuuuggugacu....................	2	1	Fm3
........................................................................Naucacagucugcuuuggugacu....................	9	1	Fm3
........................................................................uaucacagucGgcuuuggugacuu...................	1	1	Fm3
```

Star Mature

```
ccacaccuugcucuucuccuacgugaccaucgagccaccaaagugacuguggugug uuuuauaucgcucauaucacagucugcuuuggugacuugaauguguucacucaaacc
......................................................................uaucacagucugcuuuggugacCu...................   3     1   Fm3
......................................................................uaucacagucugcuuuggugacuu...................   110   0   Fm3
......................................................................uaucacagucugcuuuggugacuA...................   2     1   Fm3
......................................................................uaucacagucugcuuugUugacuu...................   1     1   Fm3
......................................................................uaucacagucugcuuCggugacuu...................   1     1   Fm3
......................................................................uaucacagucugcuuuggugacuG...................   21    1   Fm3
......................................................................uaucacagucugcuuuAgugacuu...................   1     1   Fm3
......................................................................uaucacagucugcuuuggugacuAg..................   2     1   Fm3
......................................................................uaucacagucugcuuuggugacuuC..................   1     1   Fm3
.......................................................................aucacagucuUcuuuggugacu....................   1     1   Fm3
........................................................................ucacagucugcuuuggugacu....................   2     0   Fm3
...........................................................................cagucugcuuuggugacu....................   3     0   Fm3
...........................................................................cagucugcuuuggugacG....................   1     1   Fm3

......................................................................uaucacagucugcuuugg.........................   2     0   Mm2
......................................................................uaucacagucugcuuuggu........................   6     0   Mm2
......................................................................uaucacagucugcuuuggA........................   1     1   Mm2
......................................................................uaucacagucugcuuugguA.......................   3     1   Mm2
......................................................................uaucacagucugcuuugguU.......................   5     1   Mm2
......................................................................uaucacagucugcAuuggug.......................   1     1   Mm2
......................................................................uaucacagucugcuuuggug.......................   24    0   Mm2
......................................................................uaucacagucugcuuuUguga......................   1     1   Mm2
......................................................................uaucacagCcugcuuugguga......................   1     1   Mm2
......................................................................uaucacagucugcuuuggugU......................   2     1   Mm2
......................................................................uaucacagucAgcuuugguga......................   1     1   Mm2
......................................................................uaucacagucugcuuugUuga......................   1     1   Mm2
......................................................................uaucacagucugcuuugguga......................   90    0   Mm2
......................................................................uaucacagucugcuuugAuga......................   2     1   Mm2
......................................................................uaucacagucugcuuuggugG......................   15    1   Mm2
......................................................................uaucacagucugcuuugguAac.....................   1     1   Mm2
......................................................................uaucacagucGgcuuuggugac.....................   1     1   Mm2
......................................................................uaucacagucugcuuuggugaU.....................   2     1   Mm2
......................................................................Caucacagucugcuuuggugac.....................   2     1   Mm2
......................................................................uaucacagucuUcuuuggugac.....................   1     1   Mm2
......................................................................uaucaUagucugcuuuggugac.....................   1     1   Mm2
......................................................................uaucacagucugUuuuggugac.....................   1     1   Mm2
......................................................................uaucacagucugcuuugCugac.....................   1     1   Mm2
......................................................................uaucacagucugcuuugAugac.....................   3     1   Mm2
......................................................................uaucacagucugcuuuggugac.....................   466   0   Mm2
......................................................................uaucacagucugcuuuggugaA.....................   1     1   Mm2
......................................................................uaucacagucugcuuuCgugac.....................   1     1   Mm2
......................................................................uaucacagucAgcuuuggugacu....................   4     1   Mm2
......................................................................uaucacagucugcuuugAugacu....................   14    1   Mm2
......................................................................uaucacagucugcuuugCugacu....................   13    1   Mm2
......................................................................uaucacNgucugcuuuggugacu....................   1     1   Mm2
......................................................................uaucacagucugcuAuggugacu....................   1     1   Mm2
......................................................................uaucacagucugcuuuggugacA....................   9     1   Mm2
......................................................................uaucacGgucugcuuuggugacu....................   3     1   Mm2
......................................................................uauUacagucugcuuuggugacu....................   1     1   Mm2
......................................................................uaucacagucugcuuuggugacC....................   4     1   Mm2
......................................................................uauAacagucugcuuuggugacu....................   1     1   Mm2
......................................................................uaucacagucugcuuuggugUcu....................   1     1   Mm2
......................................................................uaucacagucugcuuuggAgacu....................   1     1   Mm2
......................................................................uaucacagCcugcuuuggugacu....................   1     1   Mm2
......................................................................uaucacagucCgcuuuggugacu....................   2     1   Mm2
......................................................................uaucacaUucugcuuuggugacu....................   1     1   Mm2
......................................................................uaucacagucGgcuuuggugacu....................   4     1   Mm2
......................................................................uaucaUagucugcuuuggugacu....................   1     1   Mm2
......................................................................uUucacagucugcuuuggugacu....................   1     1   Mm2
......................................................................uaucacUgucugcuuuggugacu....................   1     1   Mm2
......................................................................uaucacagucugcuuugUugacu....................   10    1   Mm2
......................................................................uaucacagucugcuuuAgugacu....................   6     1   Mm2
......................................................................uaucacagucugcuuuggugacG....................   95    1   Mm2
......................................................................uaucCcagucugcuuuggugacu....................   1     1   Mm2
......................................................................uaucacagucugcuuuggugacu....................   2738  0   Mm2
......................................................................uaucacagucugcuuuCgugacu....................   11    1   Mm2
......................................................................uaucacagucugcGuuggugacu....................   1     1   Mm2
......................................................................Naucacagucugcuuuggugacuu...................   1     1   Mm2
......................................................................uaucacagucugcuuuggugacuA...................   1     1   Mm2
......................................................................uaucacagucugcuuuggugacuu...................   60    0   Mm2
```

```
                                  Star                                   Mature
ccacaccuugcucuucuccuacgugaccaucgagccaccaaagugacuguggugugguuuuauaucgcucauaucacagucugcuuuggugacuugaauguguucacucaaacc
......................................................................uaucacGgucugcuuuggugacuu...................   1     1   Mm2
......................................................................uaucacagucugcuuuggugacuG...................   3     1   Mm2
......................................................................uaucacagucugcuuuUgugacuu...................   1     1   Mm2
......................................................................uaucacagucugcuuuggugacuuU..................   7     1   Mm2
...........................................................................cagucugcuuuggugacu....................   1     0   Mm2

..................................ccaccaaagugacuguggugug.........................................................   7     0   Mm1
......................................................................uaucacagucugcuuuggu........................   12    0   Mm1
......................................................................uaucaUagucugcuuuggug.......................   1     1   Mm1
......................................................................uaucacagucugcuuuggug.......................   8     0   Mm1
......................................................................uaucacagucugcuuugUuga......................   1     1   Mm1
......................................................................uaucacagucugcuuugguga......................   46    0   Mm1
......................................................................uaucacagucugcAuuggUga......................   1     1   Mm1
......................................................................uaucacagucAgcuuuggUga......................   1     1   Mm1
......................................................................uaucacagucugcuuuAguga......................   1     1   Mm1
......................................................................uaucacagucugcuuuggugU......................   9     1   Mm1
......................................................................uaucacagucugcuuugAugac.....................   2     1   Mm1
......................................................................uaucacagucugcuuuggugac.....................   225   0   Mm1
......................................................................uaucacagucugcuuugUugac.....................   3     1   Mm1
......................................................................uaucacagucugcuuugCugac.....................   2     1   Mm1
......................................................................uaucacagucGgcuuuggugacu....................   1     1   Mm1
......................................................................uaAcacagucugcuuuggugacu....................   1     1   Mm1
......................................................................uaucacagucugcuuuCgugacu....................   13    1   Mm1
......................................................................uaucacagucugcuuuAgugacu....................   4     1   Mm1
......................................................................uauUacagucugcuuuggugacu....................   1     1   Mm1
......................................................................uaucacagucugcuuugguUacu....................   2     1   Mm1
......................................................................uaucacagucAgcuuuggugacu....................   5     1   Mm1
......................................................................uaucacagucugcuuuggAgacu....................   1     1   Mm1
......................................................................uaucacagucugcuuAggugacu....................   1     1   Mm1
......................................................................uGucacagucugcuuuggugacu....................   1     1   Mm1
......................................................................uaucacagucugcuuugUugacu....................   16    1   Mm1
......................................................................uaucacagucugcuuuggugacu....................   2236  0   Mm1
......................................................................uaucacagucCgcuuuggugacu....................   2     1   Mm1
......................................................................uaucacGgucugcuuuggugacu....................   4     1   Mm1
......................................................................uaucacagucugcuuugAugacu....................   15    1   Mm1
......................................................................uaucacagucugcCuuggugacu....................   1     1   Mm1
......................................................................uaucacagucugcuuugCugacu....................   15    1   Mm1
......................................................................uaucacagucugcuuuggugacG....................   14    1   Mm1
......................................................................uaucacagucugcuuuggugUcu....................   1     1   Mm1
......................................................................uaucacagucugcuuuUgugacu....................   2     1   Mm1
......................................................................uaucacagucugcuuugUugacuu...................   1     1   Mm1
......................................................................uaucacagucugcuuuggugacuu...................   44    0   Mm1
......................................................................uaucacagucugcuuugAugacuu...................   3     1   Mm1
......................................................................uaucacagucugcuuuggugacuG...................   5     1   Mm1
......................................................................uaucacagucugcuuuggugacuuU..................   7     1   Mm1

..................................ccaccaaagugacuguggugug.........................................................   3     0   Fm1
..................................ccaccaaagugacugugguguggC........................................................   4     1   Fm1
..................................ccaccaaagugacuguggugugguuuuauaucAcuc............................................   1     1   Fm1
........................................aagugacugugguguguuuuaua..................................................   3     0   Fm1
.....................................................................Cuaucacagucugcuuuggugacu....................   1     1   Fm1
......................................................................uaucacagucugcuuugg.........................   8     0   Fm1
......................................................................uaucacagucugcuuuggu........................   15    0   Fm1
......................................................................uaucacagucugcuuugAu........................   2     1   Fm1
......................................................................uaGcacagucugcuuuggu........................   1     1   Fm1
......................................................................uaucacNgucugcuuuggu........................   1     1   Fm1
......................................................................uaucacaUucugcuuuggug.......................   1     1   Fm1
......................................................................uaucacagucugcuuugguU.......................   9     1   Fm1
......................................................................uaucacagucugcuuugguA.......................   2     1   Fm1
......................................................................uaucacagucugcuuugAug.......................   4     1   Fm1
......................................................................uaucacagucugcuuuggug.......................   67    0   Fm1
......................................................................uaucacagucugcuuugCug.......................   2     1   Fm1
......................................................................uaucacagucugcuuuUgug.......................   1     1   Fm1
......................................................................uaucacagucugcuuugguga......................   94    0   Fm1
......................................................................uaucacagucugcuuuggugG......................   28    1   Fm1
......................................................................uaucacagucugcuuugAuga......................   7     1   Fm1
......................................................................uaucacagucugcuuuggugU......................   3     1   Fm1
......................................................................uaucacagucugcuuugguUac.....................   1     1   Fm1
......................................................................uaucacagucugcuuuUgugac.....................   2     1   Fm1
......................................................................uaucacagucugcuuuCgugac.....................   2     1   Fm1
```

```
                                                   Star                                Mature
ccacaccuugcucuucuccuacgugaccaucgagccaccaaagugacuguggugugguuuuauaucgcucauaucacagucugcuuuggugacuugaauguguucacucaaacc
......................................................................uaucacagucugcuuuggugac.....................    841     0    Fm1
......................................................................uaucacagucugcuuugCugac.....................    9       1    Fm1
......................................................................uaucUcagucugcuuuggugac.....................    1       1    Fm1
......................................................................uaucacagucugcuuugAugac.....................    10      1    Fm1
......................................................................uaucacagucAgcuuuggugac.....................    4       1    Fm1
......................................................................uaucacagucugcuuuggugaA.....................    4       1    Fm1
......................................................................uauAacagucugcuuuggugac.....................    1       1    Fm1
......................................................................uaucacagucugcuuuAgugac.....................    2       1    Fm1
......................................................................Aaucacagucugcuuuggugac.....................    1       1    Fm1
......................................................................uaucacagucugcuuuUugac.....................    4       1    Fm1
......................................................................uaucacagucugcuuuggCgac.....................    2       1    Fm1
......................................................................uaucacagucugcuuuggugaU.....................    2       1    Fm1
......................................................................uaucacagucGgcuuuggugac.....................    1       1    Fm1
......................................................................uaucacagucuAcuuuggugacu....................    1       1    Fm1
......................................................................uaucacagucugUuuuggugacu....................    4       1    Fm1
......................................................................uaucGcagucugcuuuggugacu....................    2       1    Fm1
......................................................................uaucacaguAugcuuuggugacu....................    2       1    Fm1
......................................................................uaucacagucugcuuuggugGcu....................    3       1    Fm1
......................................................................Caucacagucugcuuuggugacu....................    1       1    Fm1
......................................................................uaucacagucuUcuuuggugacu....................    2       1    Fm1
......................................................................uaucacagucugcuAuggugacu....................    1       1    Fm1
......................................................................uaucacagucugcuuuggugacC....................    17      1    Fm1
......................................................................uaucaUagucugcuuuggugacu....................    3       1    Fm1
......................................................................uaucacagucugcuuuUugacu....................    44      1    Fm1
......................................................................uaucacUgucugcuuuggugacu....................    2       1    Fm1
......................................................................uaucacagucAgcuuuggugacu....................    15      1    Fm1
......................................................................uUucacagucugcuuuggugacu....................    2       1    Fm1
......................................................................uaucacagucugcCuuggugacu....................    1       1    Fm1
......................................................................uaAcacagucugcuuuggugacu....................    2       1    Fm1
......................................................................uGucacagucugcuuuggugacu....................    1       1    Fm1
......................................................................uaucaNagucugcuuuggugacu....................    1       1    Fm1
......................................................................uauGacagucugcuuuggugacu....................    1       1    Fm1
......................................................................uaucacagucugcuuuUgugacu....................    13      1    Fm1
......................................................................uaCcacagucugcuuuggugacu....................    2       1    Fm1
......................................................................uaucacagucugcAuuggugacu....................    1       1    Fm1
......................................................................uaGcacagucugcuuuggugacu....................    2       1    Fm1
......................................................................uaucacagCcugcuuuggugacu....................    1       1    Fm1
......................................................................uaucacagucugcuuCggugacu....................    3       1    Fm1
......................................................................uaucacagucugcuuuggugacu....................    8971    0    Fm1
......................................................................uaucacagucGgcuuuggugacu....................    7       1    Fm1
......................................................................uaucacagucugcuuuggugaUu....................    2       1    Fm1
......................................................................uauUacagucugcuuuggugacu....................    2       1    Fm1
......................................................................uaucacagucugcuuuggAgacu....................    2       1    Fm1
......................................................................uaucacaguUugcuuuggugacu....................    9       1    Fm1
......................................................................uaucacagucugcuuuCgugacu....................    27      1    Fm1
......................................................................uaucacagucCgcuuuggugacu....................    8       1    Fm1
......................................................................uaucacagucugcuuugguUacu....................    1       1    Fm1
......................................................................uaucacagucugcuuugAugacu....................    59      1    Fm1
......................................................................uaucacagucugcuuugguAacu....................    2       1    Fm1
......................................................................Naucacagucugcuuuggugacu....................    3       1    Fm1
......................................................................uauAacagucugcuuuggugacu....................    1       1    Fm1
......................................................................uaucacGgucugcuuuggugacu....................    5       1    Fm1
......................................................................uaucacaUucugcuuuggugacu....................    1       1    Fm1
......................................................................uaucacagucugcuuuggugacA....................    15      1    Fm1
......................................................................uaucacagucugAuuuggugacu....................    1       1    Fm1
......................................................................uaucacagucugcuuugCugacu....................    57      1    Fm1
......................................................................uaucacagucugcuuuAgugacu....................    16      1    Fm1
......................................................................uaucacNgucugcuuuggugacu....................    1       1    Fm1
......................................................................Aaucacagucugcuuuggugacu....................    2       1    Fm1
......................................................................uaucCcagucugcuuuggugacu....................    3       1    Fm1
......................................................................uaucacagucugcGuuggugacu....................    2       1    Fm1
......................................................................uaucacagucugcuuuggugacG....................    360     1    Fm1
......................................................................uaucacagucugcuuuAgugacuu...................    1       1    Fm1
......................................................................uaucacagucugcuuuggugacuC...................    14      1    Fm1
......................................................................uaucacagucugcuuuUgugacuu...................    1       1    Fm1
......................................................................uaucacagucAgcuuuggugacuu...................    1       1    Fm1
......................................................................uaucacagucugcuuuggugacuG...................    2       1    Fm1
......................................................................uaucacagucugcuuugUugacuu...................    2       1    Fm1
......................................................................uaucacagucugcuuugguUacuu...................    1       1    Fm1
......................................................................uaucacagucugcuuuggugacuu...................    175     0    Fm1
```

Star   Mature

```
ccacaccuugcucuucuccuacgugaccaucgagccaccaaagugacugugguguguuuuauaucgcucauaucacagucugcuuuggugacuugaauguguucacucaaacc
......................................................................uaucacagucugcuuuggugacuuU..................   26     1    Fm1
.......................................................................aucacagucugcuuuggugacu....................   1      0    Fm1
.......................................................................aucacagucugcuuuggugUcu....................   1      1    Fm1
..........................................................................acagucugcuuuggugac.....................   3      0    Fm1
..........................................................................acagucugcuuuggugacu....................   2      0    Fm1

......................................................................uaucacagucugcuuuggG........................   3      1    Fm2
......................................................................uaucacagucugcuuuggu........................   12     0    Fm2
......................................................................uaucacagucugcuuuggug.......................   21     0    Fm2
......................................................................uaucacagucugcuuugCug.......................   1      1    Fm2
......................................................................uaucacagucugcuuugguA.......................   1      1    Fm2
......................................................................uaucacagucugcuuugUuga......................   1      1    Fm2
......................................................................uaucacagucugcuuuggugа......................   84     0    Fm2
......................................................................uaucacagucugcuuuggugU......................   3      1    Fm2
......................................................................uaucacagucugcuuuUguga......................   2      1    Fm2
......................................................................uaucacagucugcuuuggugG......................   20     1    Fm2
......................................................................uaucacagCcugcuuuggugа......................   1      1    Fm2
......................................................................uaucacagucugcuuuggugac.....................   406    0    Fm2
......................................................................uaucacagucugcuuugCugac.....................   3      1    Fm2
......................................................................uaucacagucAgcuuuggugac.....................   1      1    Fm2
......................................................................uaucacagucGgcuuuggugac.....................   1      1    Fm2
......................................................................uaucacagucugcuuuAgugac.....................   1      1    Fm2
......................................................................uaucacagucugcuuugAugac.....................   1      1    Fm2
......................................................................uaucacagucugcuuugUugac.....................   3      1    Fm2
......................................................................uaucacagucugcuuuUgugac.....................   1      1    Fm2
......................................................................uaucacagucCgcuuuggugac.....................   1      1    Fm2
......................................................................uaucacagucugcuuuCgugac.....................   2      1    Fm2
......................................................................uaucacagucugcCuuggugacu....................   1      1    Fm2
......................................................................uaCcacagucugcuuuggugacu....................   1      1    Fm2
......................................................................uaucacagucugcuuuggugacG....................   168    1    Fm2
......................................................................uaucacGgucugcuuuggugacu....................   2      1    Fm2
......................................................................uUucacagucugcuuuggugacu....................   1      1    Fm2
......................................................................uaucacagucugcuuuUgugacu....................   9      1    Fm2
......................................................................uaAcacagucugcuuuggugacu....................   2      1    Fm2
......................................................................uaucacagucugcuuugCugacu....................   18     1    Fm2
......................................................................uaucacagucugcuuugguAacu....................   1      1    Fm2
......................................................................uaucacagucCgcuuuggugacu....................   2      1    Fm2
......................................................................Naucacagucugcuuuggugacu....................   2      1    Fm2
......................................................................uaucaGagucugcuuuggugacu....................   1      1    Fm2
......................................................................uaucacagucugcuuuCgugacu....................   8      1    Fm2
......................................................................uaucacagAcugcuuuggugacu....................   2      1    Fm2
......................................................................uaucacagucugcuuuAgugacu....................   6      1    Fm2
......................................................................uaucacagucuAcuuuggugacu....................   1      1    Fm2
......................................................................uaucacagucugcuuugguUacu....................   1      1    Fm2
......................................................................uGucacagucugcuuuggugacu....................   1      1    Fm2
......................................................................uaucacagucAgcuuuggugacu....................   8      1    Fm2
......................................................................uaucacagucuUcuuuggugacu....................   1      1    Fm2
......................................................................uaucacagucugcuuuggugacA....................   19     1    Fm2
......................................................................uaucacaguAugcuuuggugacu....................   1      1    Fm2
......................................................................uaucacagucugcuuuggAgacu....................   1      1    Fm2
......................................................................uaucacagucugcuuugAugacu....................   15     1    Fm2
......................................................................uaucacagucugUuuuggugacu....................   9      1    Fm2
......................................................................uaucacagucugcuuuggugacu....................   3053   0    Fm2
......................................................................uaucacagucugcuuGggugacu....................   1      1    Fm2
......................................................................uaucacaguUugcuuuggugacu....................   8      1    Fm2
......................................................................uaucacagucugcuuuggugacC....................   6      1    Fm2
......................................................................uaucacagucugcuuugguCacu....................   1      1    Fm2
......................................................................uaucacagucugcuuugUugacu....................   11     1    Fm2
......................................................................uaucacagucGgcuuuggugacu....................   4      1    Fm2
......................................................................uaucGcagucugcuuuggugacuu...................   1      1    Fm2
......................................................................uaucacagucugcuuugCugacuu...................   1      1    Fm2
......................................................................uaucacagucugcuuuggugacuu...................   68     0    Fm2
......................................................................uaucacagucugcuuuAgugacuu...................   1      1    Fm2
......................................................................uaucacagucugcuuugAugacuu...................   2      1    Fm2
```
